# Flying-Fox Roost Disturbance and Hendra Virus Spillover Risk

**DOI:** 10.1371/journal.pone.0125881

**Published:** 2015-05-27

**Authors:** Daniel Edson, Hume Field, Lee McMichael, David Jordan, Nina Kung, David Mayer, Craig Smith

**Affiliations:** 1 Queensland Centre for Emerging Infectious Diseases, Biosecurity Queensland, Department of Agriculture and Fisheries, Brisbane, Queensland, Australia; 2 EcoHealth Alliance, New York, New York, United States of America; 3 School of Veterinary Science, The University of Queensland, Gatton, Queensland, Australia; 4 Department of Primary Industries, Wollongbar, New South Wales, Australia; 5 Department of Agriculture and Fisheries, Brisbane, Queensland, Australia; Metabiota, UNITED STATES

## Abstract

Bats of the genus *Pteropus* (flying-foxes) are the natural host of Hendra virus (HeV) which periodically causes fatal disease in horses and humans in Australia. The increased urban presence of flying-foxes often provokes negative community sentiments because of reduced social amenity and concerns of HeV exposure risk, and has resulted in calls for the dispersal of urban flying-fox roosts. However, it has been hypothesised that disturbance of urban roosts may result in a stress-mediated increase in HeV infection in flying-foxes, and an increased spillover risk. We sought to examine the impact of roost modification and dispersal on HeV infection dynamics and cortisol concentration dynamics in flying-foxes. The data were analysed in generalised linear mixed models using restricted maximum likelihood (REML). The difference in mean HeV prevalence in samples collected before (4.9%), during (4.7%) and after (3.4%) roost disturbance was small and non-significant (P = 0.440). Similarly, the difference in mean urine specific gravity-corrected urinary cortisol concentrations was small and non-significant (before = 22.71 ng/mL, during = 27.17, after = 18.39) (P= 0.550). We did find an underlying association between cortisol concentration and season, and cortisol concentration and region, suggesting that other (plausibly biological or environmental) variables play a role in cortisol concentration dynamics. The effect of roost disturbance on cortisol concentration approached statistical significance for region, suggesting that the relationship is not fixed, and plausibly reflecting the nature and timing of disturbance. We also found a small positive statistical association between HeV excretion status and urinary cortisol concentration. Finally, we found that the level of flying-fox distress associated with roost disturbance reflected the nature and timing of the activity, highlighting the need for a ‘best practice’ approach to dispersal or roost modification activities. The findings usefully inform public discussion and policy development in relation to Hendra virus and flying-fox management.

## Introduction

Bats of the genus *Pteropus*, commonly known as flying-foxes, are the natural host of Hendra virus (HeV), a novel paramyxovirus that periodically causes fatal disease in horses and consequently humans in Australia [1–3]. HeV was first reported in 1994 associated with an outbreak of acute and highly pathogenic respiratory disease in a horse stable complex in Brisbane, Australia [4]. In the ensuing years to 31 December 2014, there have been 92 confirmed or suspect equine cases [5], seven confirmed human cases [3] and two confirmed canine cases [6] in the adjoining eastern Australian states of Queensland and New South Wales. The evident route of equine infection is oro-nasal, with contaminated pasture, feed or surfaces most plausible [7]. All human cases have had close contact with the body fluids of infected horses [3,8–10]. A serologic survey of persons occupationally or recreationally exposed to flying-foxes found no evidence of infection [11]. Flying-foxes are nomadic mammals that forage nightly on blossoms and/or fruit, and roost daily in colonies that can number hundreds to hundreds of thousands of individuals. There are four species in mainland Australia: *Pteropus alecto* (Black flying-fox), *P*. *poliocephalus* (Grey-headed flying-fox), *P*. *conspicillatus* (Spectacled flying-fox) and *P*. *scapulatus* (Little red flying-fox) [12–14].

In recent decades, the number of flying-fox roosts in urban areas, and the frequency of occupation of these roosts, has dramatically increased, likely reflecting both an expanded urban footprint and food resource availability in urban areas [15]. While an evident demographic ‘urban shift’ has been reported in grey-headed [16], black [17] and spectacled flying-foxes [18,19], the urban presence of little red flying-foxes is typically episodic, reflecting their highly nomadic life history trait [12]. Regardless of the underlying ecological drivers of this greater human—flying-fox interface, the increased urban presence of flying-foxes often provokes negative sentiments from nearby residents and some members the broader community. Complaints generally instance reduced social amenity (objectionable noise, soiling and smell) and health concerns (primarily perceived HeV exposure risk), the latter notwithstanding the absence of evidence of direct flying-fox to human transmission [20]. Paradoxically, it is the transient presence of little red flying-foxes that tends to provoke more public angst because of their typically large numbers and tree-damaging dense roosting habits. Overall, the situation has resulted in increased calls for more active flying-fox management, and the dispersal of urban colonies [21]. Juxtaposed with this perspective is the hypothesis that disturbance of flying-foxes may result in a stress-mediated increase in virus excretion, infection, translocation and transmission, and an increased HeV exposure risk for horses and thus humans. This thinking reflects the findings of Plowright et al. (2008), who found a correlation between increased HeV antibody prevalence in *P*. *scapulatus* and nutritional and reproductive stress [22]. Glucocorticoid hormones such as cortisol and corticosterone are key regulators of energy balance in mammalian species, and elevations in these hormones can indicate stress [23,24], but objective studies of glucocorticoid hormone dynamics and physiological stress in free-living flying-foxes (and wildlife generally) are limited, reflecting fundamental methodological challenges [25–27]. However, McMichael et al. (2014) have recently described a robust approach to measuring cortisol values in flying-fox populations [28].

Studies specifically examining flying-fox roost disturbance and its impact on HeV infection dynamics are lacking. In this study, we seek to address this knowledge gap and provide a more objective basis for policy development. Our primary objective was to identify any temporal association between flying-fox roost disturbance and HeV excretion prevalence. Secondary objectives were to examine the relationship between roost disturbance and cortisol concentration as a quantifiable stress parameter in flying-foxes, and the relationship between HeV excretion and cortisol concentration.

## Methods

### Study design and roost selection

The study design incorporated elements of the Before-After-Control-Impact (BACI) approach used to detect and quantify putative anthropogenic environmental impacts [29], and sought to sample as many spatial and temporal scales as practically possible. Roosts were enrolled in the adjoining Australian states of Queensland and New South Wales between September 2011 and November 2012. The main inclusion criterion for primary roosts was that a damage mitigation permit (DMP) (or equivalent) for roost modification or dispersal had been granted or sought from the (then) Queensland Department of Environment and Resource Management, the (subsequent) Queensland Department of Environment and Heritage Protection, or the New South Wales Department of Environment and Heritage. DMP applications were typically submitted by local councils or resident groups. The enrolment of primary roosts occurred progressively over the study period as DMP applications were received and considered by the state environmental authorities. An additional inclusion criterion was that the roost was accessible for the under-roost collection of pooled urine samples and for the capture of individual flying-foxes for telemetry studies (data not presented here). Where possible, primary roosts were sampled on a monthly basis prior to the commencement of permitted disturbance activities, with more frequent sampling (weekly—fortnightly) during and after disturbance events. Putative post-disturbance roosts, based on spatial proximity to at least one primary roost and/or telemetry data (Edson *et al*, 2012 in preparation; John Martin and Justin Welbergen, pers. comm. 2012), were also progressively enrolled, and defined as ‘secondary roosts’. Secondary roosts were typically sampled opportunistically in both the pre-, during- and post-dispersal periods. Samples collected from primary and secondary roosts at time points either prior to the commencement of permitted disturbance, or after 56 days post-cessation of permitted disturbance (see below) contributed to baseline data.

### Sampling strategy

Pooled urine samples were collected from underneath roosting flying-foxes before, during and after the permitted disturbance where logistically possible. The collection of pooled urine samples was adapted from Field et al (2011) [30]. Briefly, plastic sheeting measuring 3.6 m x 2.6 m was placed under trees in which flying-foxes were roosting, typically pre-dusk. Colony-level data including size, species composition, and reproductive status were recorded. The following morning at dawn, pooled urine samples were collected from each sheet using a graduated micropipette and 1 mL filter tip, placed in a graduated screw-cap 2 mL micro cryotube, and held on ice bricks. The target sample size and volume was 30 x 1.25 mL, with typically three or four pooled samples from each of ten sheets. Pooled samples were methodically collected from discrete sections of each sheet to minimise an individual bat's potential contribution to multiple pools, and typically consisted of 10–20 discrete urine droplets. Where more than four pooled samples were collected from a sheet, four samples were randomly selected for inclusion in the analysis (using a random number generator) to maintain consistent sampling intensity across sheets. After sample collection, duplicate 50 μL samples of urine were each added to 130 μL of MagMax lysis solution (Catalogue number AM8500) to inactivate virus particles and preserve RNA for PCR testing, with the remaining sample maintained neat for cortisol assay. Samples were packed according to IATA-approved protocols, pending overnight shipment to the Queensland Department of Agriculture Forestry and Fisheries Biosecurity Sciences Laboratory (BSL) in Brisbane. Personal protective equipment during field work typically consisted of overalls or cover shirt and pants, hat with head torch, gumboots and double nitrile gloves. Equipment was routinely decontaminated with Virkon; waste was similarly decontaminated and bagged and disposed of at BSL, or where impractical, at a licensed waste management facility.

### Laboratory testing

#### Hendra virus detection

Urine samples underwent total nucleic acid extraction in the BSL Physical Containment Level 3 (PC3) laboratory using the Kingfisher automated extraction system and the MagMax viral RNA Isolation Kit (Catalogue number AMB18365) according to manufacturer’s instructions. RNA extracts (5 μL) were added to 20 μL reaction mix (AgPath-ID One-Step RT-qPCR Kit (Life Technologies)) and assayed for HeV genome using a quantitative TaqMan RT-PCR targeting the HeV M gene [31] using the Applied Biosystems 7500 Fast Real Time PCR System and AB7500 software version 2.0.6. Duplicate urine samples in lysis buffer were stored at -80°C for additional testing if required. PCR-positive samples were virus-inactivated prior to undertaking cortisol assay by addition of 0.5% Tween 20 and 0.5% Triton X-100, and incubated 30 minutes at 56°C [32]. A trial comparison of treated and untreated samples showed no difference in cortisol concentration measurements.

#### Cortisol measurement

Cortisol assays were performed in the BSL Physical Containment Level 2 (PC2) laboratory using a Caymen Chemical Company Cortisol Enzyme Immunoassay (EIA) (Product number 500360) as described by McMichael *et al*. (2014) [28]. Briefly, duplicate pooled urine samples were diluted 1:10 in the kit assay buffer, and assayed for cortisol according to manufacturer’s instructions. Cortisol concentration was determined by analysis of absorbance values using MyAssays Analysis Software Solutions ‘Four Parameter Logistic Curve’ online data analysis tool http://www.myassays.com/four-parameter-logistic-curve.assay. Urine specific gravity (USG) was measured for all urine samples using a hand-held clinical refractometer, and cortisol concentrations corrected according to mean species USG as described by McMichael *et al*. (2014) [28].

### Qualitative assessment of disturbance impact

Data on the nature and effect of permitted disturbance activities was collected by the authors or professional colleagues (see Acknowledgements), and documented by photographic, video and written records. Anecdotal reports on unpermitted activities were also received from community members who witnessed such activities. A qualitative assessment of flying-fox distress following roost disturbance was made, based on behavioural parameters including agitation, confusion, vocalisation, day-time flight, and reluctance to re-roost. A probabilistic matrix was used to assess the combination of the magnitude and frequency of activities. Magnitude was categorised as low (causing movement of a small number of animals in a discrete part of the roost), moderate (causing movement of a significant number of animals which resettle within minutes), high (causing movement and obvious distress to a significant number of animals which take a long time to settle or move to alternative roost trees/site) and extreme (causing movement of a significant number of animals throughout the entire roost and risk of death to individuals). Frequency was categorised as low (a one-off event), moderate (intermittent events over a period of time), and high (a daily event over a period of time).

### Statistical analyses

Roost and sampling event data were recorded on field data sheets and subsequently entered into an Excel spreadsheet. Hendra virus PCR and cortisol assay results were added as the results became available. The data were analysed in generalised linear mixed models (GLMM) using restricted maximum likelihood (REML) in GenStat (2013) [33]. For the model with the binary Hendra virus data as dependent variable, the errors were assumed to be binomially distributed and a logit link function was applied [34]. For the model with the cortisol concentration as dependent variable, the latter was first log transformed to address skewness and variance heterogeneity, and then modelled assuming normal errors and an identity link function, with the adjusted model means being directly back-transformed to provide geometric means. Error bars (means ± one standard error) were calculated on the log-scale (cortisol concentrations) or logit scale (HeV RNA detection), and directly back-transformed. Both Type I (sequential addition of terms to the fixed model) and Type III testing (backwards elimination of terms from the full fixed model) were utilised in model selection, and Type III probability-levels are quoted. Factors were added or excluded from the models on the basis of the change in model deviance. Where interactions could not be estimated at all, or if they could only be tested with minimal degrees of freedom, they were excluded. The following ‘fixed effect’ factors were used in the analysis: ‘Region’ defined the location of roosts that can be grouped according to their geographic proximity. Regions may constitute a single roost or multiple roosts. Where a region is comprised of multiple roosts, this consists of a primary roost subject to a permitted disturbance, and monitored secondary roosts known to receive, or putatively receiving, flying-foxes from the primary roost post-disturbance; ‘Disturbance’ indicates the timing of sample collection in relation to the disturbance treatment, with ‘before’ denoting all samples collected prior to the commencement of disturbance, ‘during’ denoting all samples collected during the period of disturbance, and ‘after’ denoting all samples collected from the disturbed roost or from known or putative destination roosts for 56 days from the cessation of disturbance; ‘Species mix’ identified the flying-fox species present in the roost: black (‘b’), grey-headed (‘g’), little red (‘r’) or spectacled (‘s’) flying-foxes, and combinations thereof. The data for levels b, g, r, s, bg and br were all substantial (200 to 600 samples per level) and so retained. To minimise possible over-parameterisation of models, the minor species composites were pooled as follows: rs into r (30 samples; 90% r and 10% s); bgr into br (30 samples, 30% b, 10% g, 60% r); and bgrs into bg (48 samples; 85% b, 13% g, 1.8% r, 0.2% s); ‘Season’ consisted of summer (December—February), autumn (March—May), winter (June—August) and spring (September—November). Month and year variables were not used as temporal variables because this resulted in an unacceptable degree of aliasing (confounding) with other factors, especially ‘disturbance’, the key variable of interest. Interactions comprised of the above were constructed for evaluation in the models, with the awareness that the extent of aliasing in observational studies can result in model over-parameterisation with the inclusion of interactions. Random effects terms for the mixed model analysis comprised ‘Sampling date’, representing calendar date of collection, and ‘samples within dates’ representing samples collected separately on a given date. Adjusted means (standardised for all terms in the model) are presented throughout. Two roosts (Yeppoon and Great Keppel Island) were excluded from the analysis, the former because the samples were rain-affected, and the latter because it was the sole evening collection, precluding valid cortisol concentration comparison with the other collections. Samples from one Sydney collection were also rain-affected and excluded from the analysis.

### Animal ethics

The collection of pooled urine samples from underneath roosting flying-foxes and the capture of individual flying-foxes for telemetry studies (data not presented here) was conducted under the (then) Department of Employment, Economic Development and Innovation Animal Ethics Committee Permit SA 2011/12/375, Queensland Environmental Protection Agency Scientific Purposes Permit WISP05810609, Queensland Department of Environment and Resource Management Scientific Purposes Permit WISP05810609, New South Wales Office of Environment and Heritage Animal Ethics Committee Permit 120206/02, New South Wales Office of Environment and Heritage Scientific Licence SL 100537.

## Results

**Fig 1:**
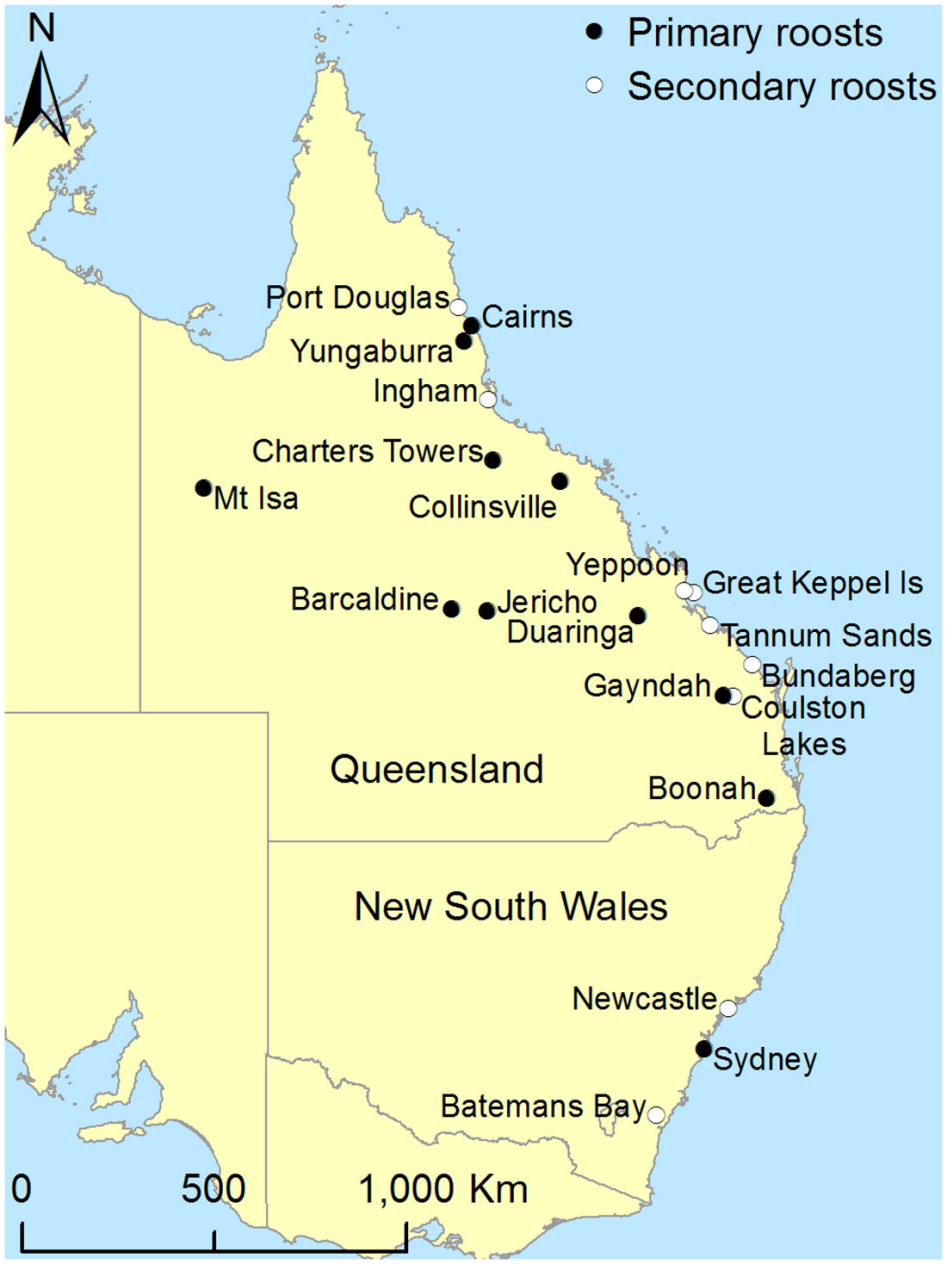
Eleven primary roosts and ten secondary roosts monitored in the eastern Australian states of Queensland and New South Wales between September 2011 and November 2012. [Sydney has two roosts—Sydney Royal Botanic Gardens (RBG), a primary roost, and Sydney Centennial Park (CP), a secondary roost. The former is indicated.]

A total of 21 roosts were enrolled over the 15-month study period, and included 11 primary roosts and 10 secondary roosts (Fig 1, Table 1). The primary roosts comprised seven roosts for which DMPs were approved, one roost for which a DMP was not approved, one roost for which a DMP application was withdrawn, and two roosts for which a DMP application outcome was still pending at the study end-date (S1 Table). Permitted disturbance activities occurred at five enrolled roosts in Queensland (n = 4) and New South Wales (n = 1). One Queensland roost (Gayndah) was subject to permitted disturbance in 2011 and again in 2012, making a total of six disturbance events. No disturbance occurred at the other two DMP-approved Queensland roosts within the study period as the animals moved of their own volition prior to the planned disturbance. A total of 91 sampling events (57 on primary roosts and 34 on secondary roosts) were undertaken, yielding 2719 pooled urine samples, and involving all four mainland Australian flying-fox species. Of the 91 sampling events, 66 (72.5%) involved the collection of baseline samples, 14 (15.4%) involved the collection of samples during a permitted disturbance event, and 11 (12.1%) involved the collection of samples after a permitted disturbance event.

**Table 1:** Relationship between 21 roosts and 18 regions^1^ monitored in the eastern Australian states of Queensland and New South Wales between September 2011 and November 2012.

Region	Roost locations	Roost type
Barcaldine	Barcaldine^2^	primary
	Collinsville^3^	secondary/primary^4^
	Mt Isa^3^	secondary
Gayndah 2011	Gayndah^2^	primary
Gayndah 2012	Gayndah^2^	primary
	Bundaberg^3^	secondary
	Coulston Lakes^3^	secondary
	Tannum Sands^3^	secondary
Sydney (RBG)	Sydney (RBG)^2^	primary
	Batemans Bay^3^	secondary
	Blackbutt^3^	secondary
	Sydney (CP) ^3^	secondary
Charters Towers	Charters Towers^2^	primary
	Ingham^3^	secondary
Duaringa	Duaringa^2^	primary
Jericho	Jericho	primary
Yungaburra	Yungaburra	primary
Cairns	Cairns	primary
Boonah	Boonah	primary
Mt Isa	Mt Isa	primary
Bundaberg	Bundaberg	secondary
Sydney (CP)	Sydney (CP)	secondary
Great Keppel Island	Great Keppel Island	secondary
Ingham	Ingham	secondary
Port Douglas	Port Douglas	secondary
Tannum Sands	Tannum Sands	secondary
Yeppoon	Yeppoon	secondary

^1^ ‘Region’ defines the geographic location of roosts for the purposes of analysis, and may constitute a single roost or multiple roosts. Roosts appearing in more than one region contributed disturbance data and baseline data at different time points.

^2^ Roosts subjected to permitted disturbance. [Sydney (RBG) = Sydney Royal Botanic Gardens.]

^3^ Roosts known to receive, or putatively receiving, flying-foxes from a disturbed roost in the same region. [Sydney (CP) = Sydney Centennial Park.]

^4^Collinsville is a primary roost based on DMP application (see S1 Table), but the sole sampling event at this roost was in the context of it putatively receiving flying-foxes following the Barcaldine roost dispersal, thus it is a secondary roost in this context.

**Fig 2:**
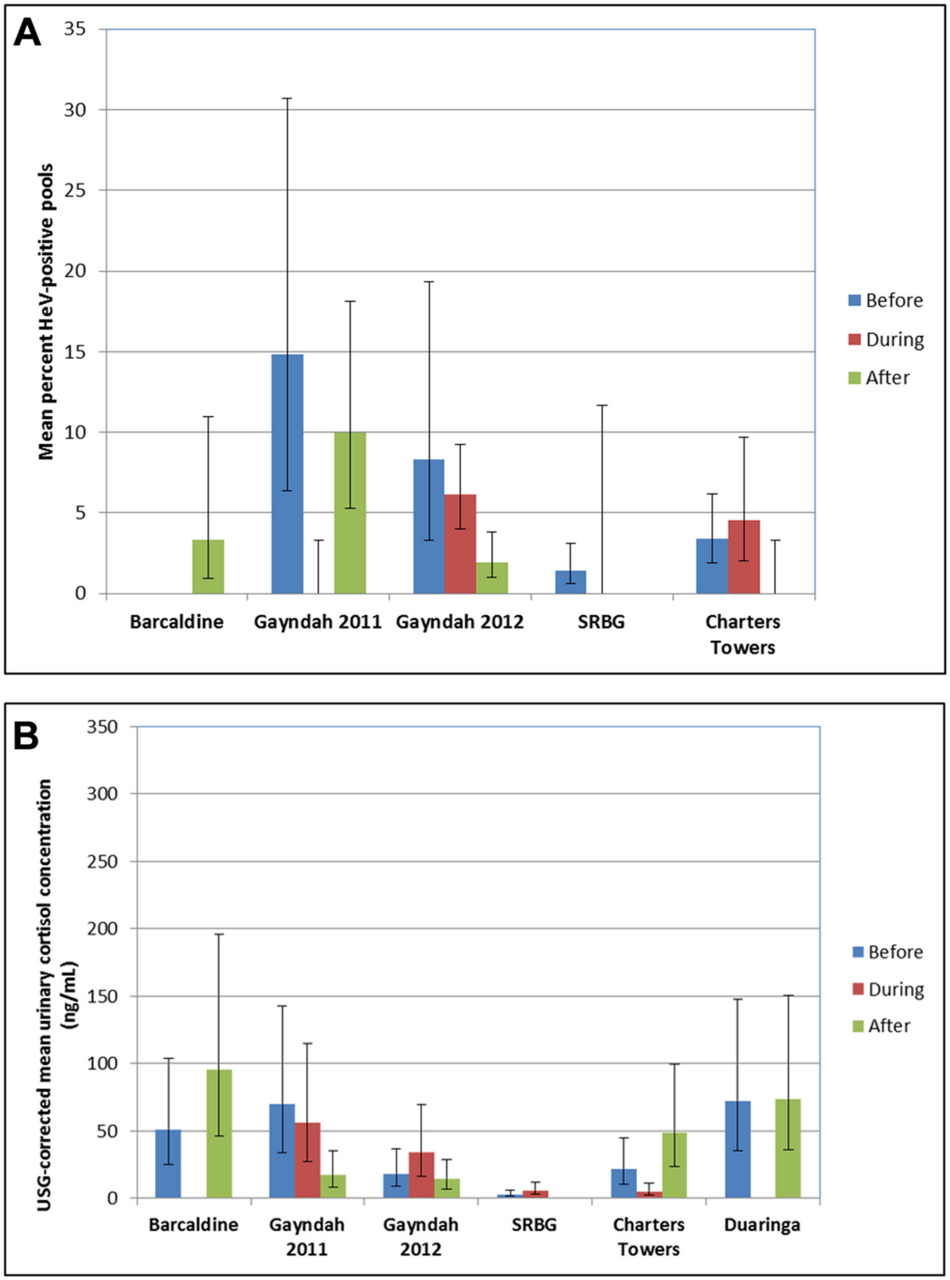
Adjusted mean HeV excretion prevalence (A) and adjusted USG-corrected urinary cortisol concentration (B) in six regions before, during and after permitted flying-fox roost disturbances in the eastern Australian states of Queensland and New South Wales between September 2011 and November 2012. Error bars represent the mean ± one standard error, obtained by back-transforming variance from the logistic scale. Approximate variance is used where HeV excretion prevalence is zero during (Gayndah 2011, SRBG) or after (Charters Towers) disturbance (A). Duaringa was excluded as HeV excretion prevalence was zero before, during and after disturbance (A). Respective baseline values using the same y-axis scale are presented in S1 Fig and S2 Fig.

**Fig 3:**
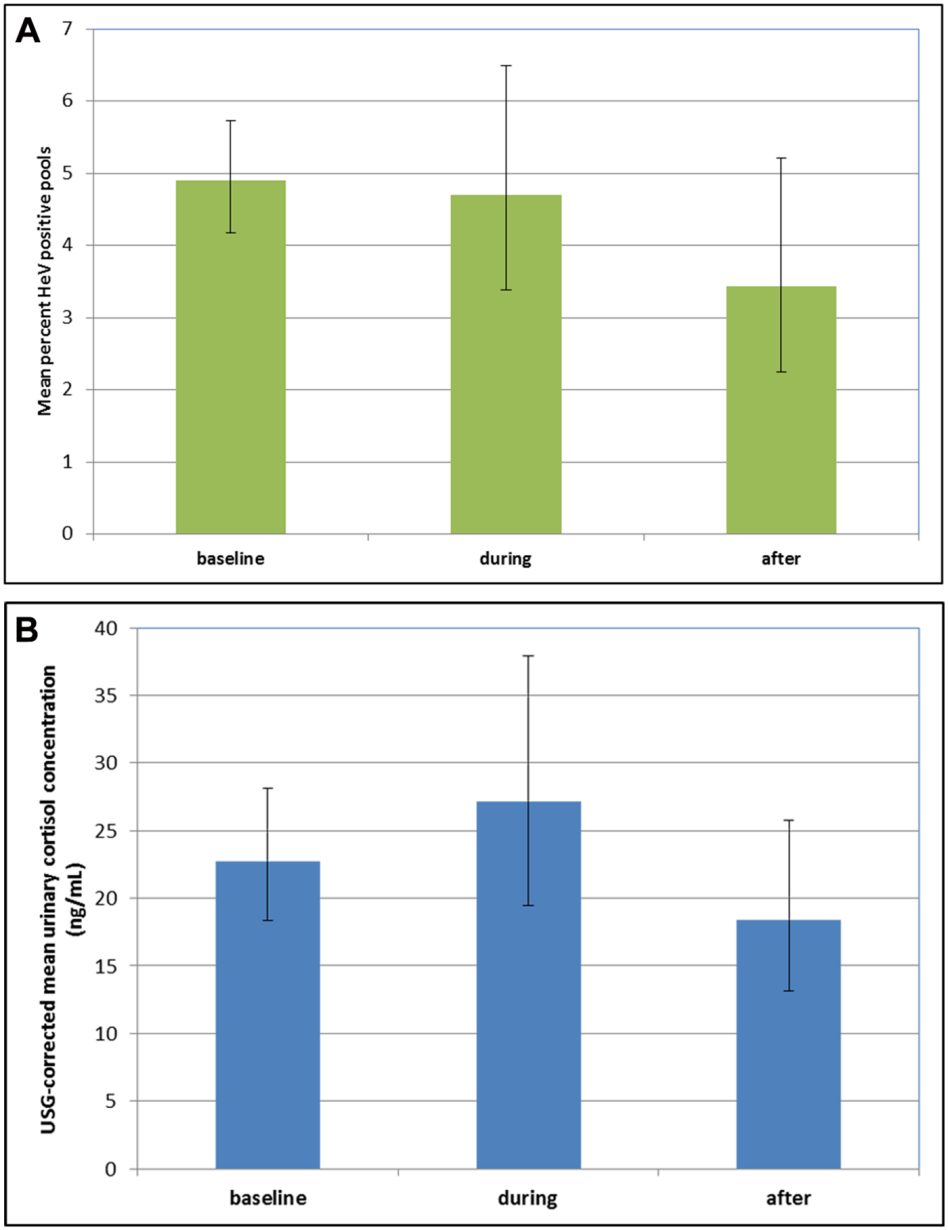
Adjusted overall effect of roost disturbance on the prevalence of HeV (A) and on USG-corrected cortisol concentration (B) in pooled urine samples from flying-foxes roosts in the eastern Australian states of Queensland and New South Wales between September 2011 and November 2012. Error bars represent the mean ± one standard error, obtained by back-transforming variance from the logistic scale.

The component data (before, during and after) for the six disturbance events are presented in Fig 2A (mean HeV excretion prevalence) and Fig 2B (USG-corrected urinary cortisol concentration). The final models for both HeV urinary excretion prevalence and for USG-corrected cortisol comprised the fixed factors ‘region’, ‘season’, ‘species mix’, ‘disturbance’ and the interaction term ‘region x disturbance’. The random effects were sampling dates, and samples within dates. The difference in overall mean HeV prevalence before (4.9%), during (4.7%) and after (3.4%) disturbance was small and non-significant (P = 0.440, Fig 3A). Similarly, the difference in overall mean USG-corrected cortisol concentrations was small and non-significant (before disturbance = 22.71 ng/mL, during disturbance = 27.17, after disturbance = 18.39) (P = 0.550, Fig 3B). The effect of disturbance on cortisol concentration approached statistical significance for region (F_7, 52_ = 1.80, P = 0.108). No other two-way interactions appeared important across various alternate models (data not shown). USG-corrected cortisol concentration varied significantly with season (F_3, 50_ = 3.91, P = 0.014) and with region (F_15, 50_ = 4.18, P < 0.001), with winter levels (34.78 ng/mL) higher than in summer (15.86 ng/mL) and autumn (18.78 ng/mL), and region levels ranging from 3.71–167.17ng/mL. Cortisol concentration varied modestly but significantly with HeV detection status (HeV negative = 15.43ng/mL, HeV positive = 21.71ng/mL) (P = 0.001), with the distribution of concentrations similar in both groups. HeV detection in single-species roosts of little red flying-foxes and grey-headed flying-foxes was always zero, hence these species were excluded from the analysis of the effect of disturbance on HeV excretion prevalence, and from the analysis of the effect of HeV detection status on cortisol concentration as they contributed no data. With these species excluded, HeV detection did not vary significantly with species mix. The baseline data showed no significant difference in the mean percentage of HeV-positive pools between regions (F_10, 63_ = 1.23, P = 0.292) (S1 Fig), but indicated significant variation in the mean adjusted urinary cortisol concentration between regions (S2 Fig).

Permitted disturbance activities ranged from the lopping and removal of trees adjacent to a roost to create a buffer, to active efforts to drive animals away from a roost (S2 Table). The latter typically involved loud noise and bright lights pre-dawn as flying-foxes returned to roost, to disrupt and dissuade roosting. At some roosts, nocturnal destruction of roost trees was used in conjunction with dispersal efforts, compounding confusion and distress when animals returned to roost. Overall, the nature, structure, timing and duration of dispersal activities, the level of dispersal monitoring, and the success of the dispersal efforts varied widely between roosts. Our qualitative assessment of flying-fox distress associated with permitted disturbance activities yielded a spectrum of impacts, ranging from low to extreme in severity, and from acute to chronic in duration. Unpermitted disturbance activities (which occurred both during and outside the periods of permitted disturbances) were more likely to provoke more severe impact and were more likely to be chronic. Aberrant behaviours such as daytime flying, circling around or near the roost, confusion, vocalization, reluctance to settle, and panting respiration and evident exhaustion in newly independent young were recorded.

## Discussion

The primary objective of the study was to identify any association between flying-fox roost disturbance and HeV excretion, with the aim of identifying whether disturbance could potentially increase the risk of HeV spillover to horses and consequently humans. To this end, there are two fundamental questions: firstly, does disturbance precipitate an increased incidence of infection in the disrupted individuals, and secondly, does disturbance promote roost connectivity and facilitate virus transmission that would otherwise not occur? Regarding the first question, we found no statistically significant association between disturbance and HeV urinary excretion prevalence, with the point estimates showing a small negative trend. Mean C_T_ values pre- and post-disturbance did not vary significantly (data not shown). Thus, while the limited number of samples per sampling event precludes identification of a trivial association, our findings demonstrate the absence of any substantial effect of roost disturbance on HeV excretion. From a HeV incident risk perspective, the latter (i.e. substantial effect) is of fundamental epidemiological relevance.

Our finding of no HeV excretion in single species roosts of little red and grey-headed flying-foxes is consistent with those of Smith et al. [35] and Goldspink et al. (in review), which suggest these species may be less efficient hosts than black or spectacled flying-foxes. If this is the case, it could be argued that the presence of little red and/or grey-headed flying-foxes in mixed roosts with black and/or spectacled flying-foxes in this study might retard HeV transmission, and so mask any effect of disturbance. We believe this is unlikely, given the dynamic proportions of each species in mixed roosts, the typical clustering of species within mixed roosts, and the number, connectivity and geographic spread of such roosts post-disturbance. However, in the absence of any single species black or spectacled flying-fox roosts being subject to permitted disturbance, we cannot exclude the possibility of a different disturbance outcome in such roosts. Regarding the second question, telemetry data shows dynamic natural ‘background’ movement of flying-foxes between roosts (Edson *et al*, 2012 in preparation; John Martin and Justin Welbergen, pers. comm. 2012). This data indicates existing strong connectivity between roosts at both regional and inter-regional levels, and suggests that sporadic anthropogenic disturbance events are unlikely to substantially alter connectivity. However, recent legislative changes to flying-fox management in Queensland (and similar legislation currently under consideration in New South Wales) which devolve the management of urban flying-fox roosts from the state environmental agency to local authorities [36,37] could result in a greater frequency of roost disturbance than in our study, with unknown effect on roost connectivity at the higher level.

The secondary study objectives were to examine the relationship between roost disturbance and cortisol concentration in flying-foxes, and between HeV excretion and cortisol concentration. We used mean pooled urinary cortisol as a quantitative measure of stress firstly because pooled urine samples can be collected under-roost without provoking a confounding stress response, and secondly because urinary cortisol is known to be a robust measure of stress in many mammalian species, correlating directly with plasma cortisol levels [28,38]. The variable seasonal and regional association between disturbance and urinary cortisol detected in this study suggests that this relationship is not fixed. Plausibly, it may reflect the nature and duration of disturbance, or alternatively or additionally, the population biology and structure of the colony. Finally, the fluctuations in urinary cortisol baseline data indicate that free-living flying-foxes are subject to periodic stressors, and suggest that the detected winter peak may reflect ‘natural’ environmental and/or physiological stressors [28,39]. A statistical association between urinary cortisol concentration and HeV detection status is not surprising given the large number of urine samples. However, the biological significance of the positive association should not be over-interpreted, firstly given the modest magnitude of the difference, and secondly because causality cannot be attributed.

While 11 primary roosts were enrolled and monitored pre-disturbance, only five were dispersed or modified within the study timeframe, and thus available for post-disturbance monitoring. This situation was a consequence of either DMP applications being withdrawn or cancelled (e.g. when the colony dispersed of its own volition prior to finalisation of the application process), approved dispersals/modifications being delayed (e.g. when dependent young were found to be present in the roost), or the non-approval of a pending DMP application, and precluded the collection of a ‘before’ and ‘after’ series of samples from more roosts. Data from primary roosts that were not disturbed contributed to baseline data. The typical presence of grey-headed flying-foxes in New South Wales (NSW) roosts and their protected status under Australian federal law [40] means that permission for dispersal in NSW typically requires approval from both federal and state environmental agencies, explaining why only one roost in NSW was approved for dispersal within the study period.

The rationale for the compilation of baseline data was to capture any natural variability in HeV excretion or urinary cortisol concentration that might occur over time or space [41]. Because the actual disturbance date was generally only made known to the researchers 48 hours in advance, sample collections were routinely undertaken weekly or fortnightly from the date of roost enrolment where logistically possible, to enable multiple collection time-points. This was not possible in Barcaldine, or Gayndah in 2011 and 2012, because of the limited time between enrolment and the commencement of disturbance, and meant fewer baseline samples from these roosts. The rationale for collecting post-disturbance from the cessation of disturbance to eight weeks post-cessation was to allow adequate time for any disturbance-related HeV transmission pulse to manifest at a regional level. Crude HeV prevalence at the sampling event level was consistent with previous studies [30].

Roost disturbance activities undoubtedly disrupt flying-fox behaviour, and Roberts et al (2011) describe issues and challenges associated with roost dispersal [42]. Our qualitative assessment in this study shows that the severity of disturbance varies in magnitude and duration, likely reflecting the nature and timing of the activity. When such activities precipitate a chaotic and/or extended flight response, cohorts that have less energetic reserves (juveniles, late pregnancy females, females with dependent young) are likely to be more severely impacted, highlighting the need for consideration of the animal welfare aspects of disturbance, particularly where the aim is to disperse the roost. With respect to the latter, the considered and consultative approach used at the Sydney Royal Botanic Gardens roost [43], largely based on the earlier Melbourne Royal Botanic Gardens dispersal [44], appears humane and effective (albeit expensive), and represents current best practice. Notwithstanding, the urinary cortisol data in this study does not suggest that dispersal or roost modification activities precipitate activation of the hypothalamic-pituitary-adrenal (HPA) axis and the development of a ‘chronic stress’ state sometimes associated with immune system dysfunction and consequent negative health states [45]. Indeed, the three roosts that had (non-significant) higher point estimates after disturbance showed at most a modest two-fold increase on pre-disturbance levels, no more than the background fluctuation in the baseline data, and orders of magnitude less than acute capture stress response in flying-foxes [28]. Nonetheless, differentiating between acute and chronic stress in wild flying-fox populations using urinary cortisol is challenging, particularly if baseline data is influenced by ‘background’ anthropogenic disturbance, and future studies might employ specific tests of HPA-axis function (such as low dose dexamethasone suppression tests or ACTH stimulation tests) that were beyond the scope of this study. Because they are volant and fundamentally nomadic, flying-foxes have some ability to avoid anthropogenic-induced stress scenarios. However, with their increasing utilisation of peri-urban and urban resources [15], flying-foxes are potentially at risk of continual harassment in the form of a rolling succession of roost disturbance or dispersal, the consequences of which are not captured by this study.

This study sought to address a knowledge gap in relation to the effect of roost disturbance on HeV excretion in an environment of polarised opinions on flying-fox management and potential HeV exposure risk. The findings, which directly measured both baseline HeV excretion and cortisol concentration values and post-disturbance values, provide a robust platform for informed public discussion and policy development in relation to Hendra virus and flying fox management in Australia. The absence of any detected increase in HeV excretion associated with roost disturbance does not negate the ‘background’ exposure risk for horses, and the need for horse-owners to adopt and maintain recommended risk management strategies [5]. The findings are also potentially equally relevant to Nipah virus, a closely related virus responsible for regular outbreaks in Bangladesh and India, and more broadly, will be of interest in parallel scenarios where urban wildlife, humans and emerging zoonoses intersect.

## Conclusions

We found no statistical association between flying-fox roost disturbance and HeV urinary excretion prevalence, indicating that roost disturbance does not precipitate increased HeV infection and excretion in dispersing flying-foxes. Further, we found no fundamental statistical association between roost disturbance and urinary cortisol concentration. We did find an underlying association between cortisol concentration and season, and cortisol concentration and region, suggesting that other (plausibly biological or environmental) variables play a role in circulating levels of cortisol. The effect of disturbance on urinary cortisol concentration approached statistical significance for region, suggesting that the relationship is not fixed, and plausibly reflecting the nature and timing of disturbance. We also found a small positive statistical association between HeV urinary excretion status and urinary cortisol concentration, but elaborating any causal association was beyond the scope of this study. Baseline data showed no significant difference in the mean percentage of HeV-positive pools between regions, but indicated significant variation in the mean adjusted urinary cortisol concentration between regions, the latter suggesting the role of other factors.

Qualitative assessment of behavioural distress associated with roost disturbance showed that the severity of impact reflected the nature and timing of the activity, and highlights the need for a ‘best practice’ approach to dispersal or roost modification activities. While the mobility of flying-foxes provides some capacity for them to escape anthropogenic disturbance, their increasing urban presence (reflecting increased urbanisation and relative food abundance) may subject them to chronic roost disturbance and harassment, the consequences of which are unknown.

## Supporting Information

S1 Fig Adjusted baseline HeV excretion prevalence (% positive pools) from six flying-fox roost regions in the eastern Australian state of Queensland between September 2011 and November 2012. Single species roosts containing either little red or grey-headed flying-foxes are excluded because of zero HeV detections in these roosts. Error bars represent the mean ± one standard error, obtained by back-transforming variance from the logistic scale. Approximate variance is used where HeV excretion prevalence is zero (Bundaberg). Y axis scales are the same as Fig 2A to facilitate direct comparison with roosts subject to permitted disturbance. [Note ‘Lakeside’ = ‘Yungaburra’].
(TIF)

S2 Fig Adjusted baseline USG-corrected mean urinary cortisol concentration (ng/ml) from 10 flying-fox roost regions in the eastern Australian states of Queensland and NSW between September 2011 and November 2012. Error bars represent the mean ± one standard error, obtained by back-transforming variance from the logistic scale. Y axis scales are the same as Fig 2B to facilitate direct comparison with roosts subject to permitted disturbance. [Note ‘Lakeside’ = ‘Yungaburra’].
(TIF)

S1 Table Geographic and DMP detail of 21 flying-fox roosts monitored in the eastern Australian states of Queensland and New South Wales between September 2011 and November 2012. (DOCX)

S2 Table Qualitative assessment of flying-fox distress associated with six permitted roost disturbance events in the eastern Australian states of Queensland and New South Wales between September 2011 and November 2012. [The Gayndah roost had permitted disturbance events in 2011 and 2012. FF = flying-foxes, P = permitted disturbance activities, NP = non-permitted disturbance activities.]
(DOCX)
