# Supplementary figures and images for: Flying-Fox Roost Disturbance and Hendra Virus Spillover Risk
Source: PLoS One. 2015 May 27;10(5):e0125881. doi: 10.1371/journal.pone.0125881 (PMC4446312; doi:10.1371/journal.pone.0125881)

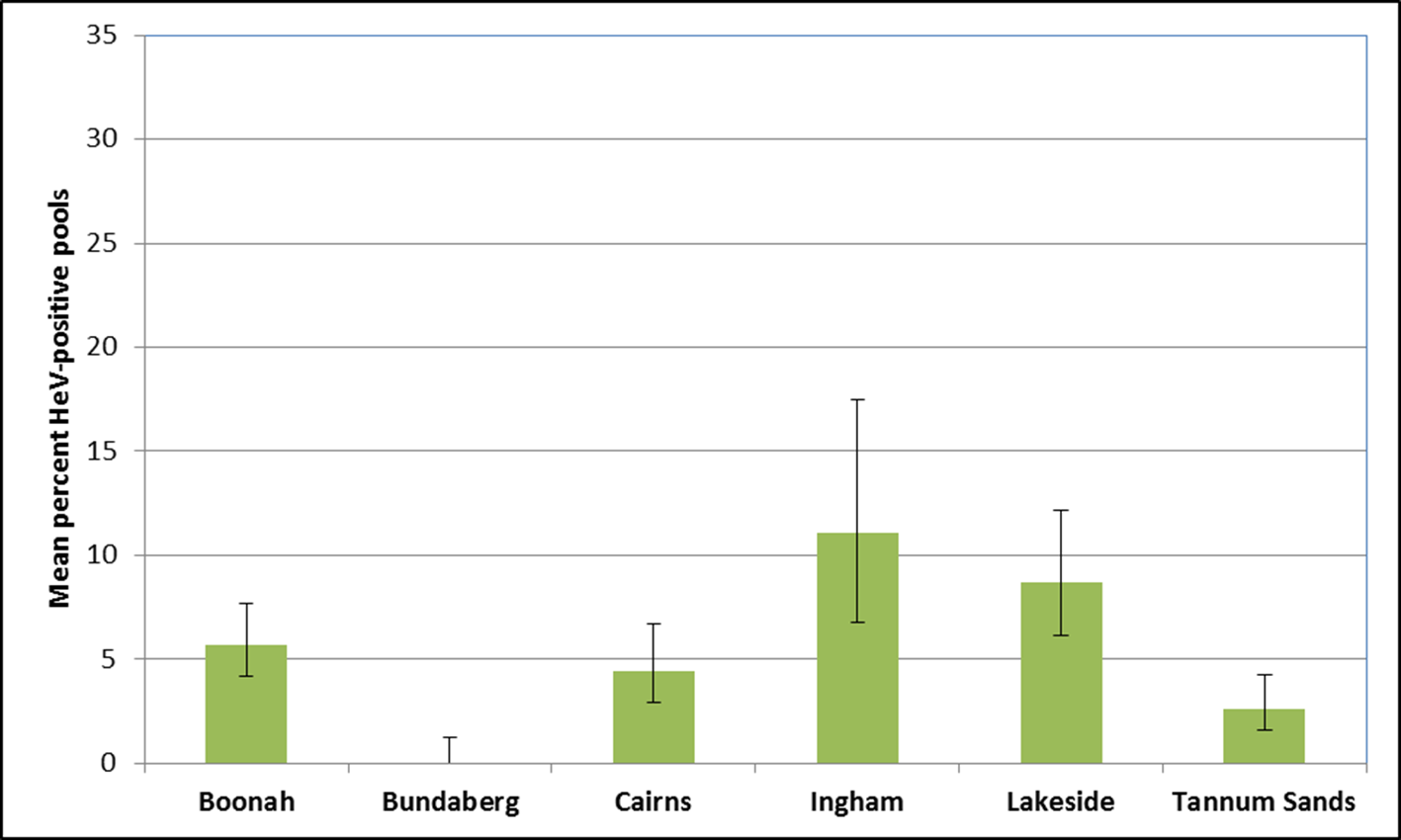

Supplement: S1 Fig — Single species roosts containing either little red or grey-headed flying-foxes are excluded because of zero HeV detections in these roosts. Error bars represent the mean ± one standard error, obtained by back-transforming variance from the logistic scale. Approximate variance is used where HeV excretion prevalence is zero (Bundaberg). Y axis scales are the same as Fig 2A to facilitate direct comparison with roosts subject to permitted disturbance. [Note ‘Lakeside’ = ‘Yungaburra’]. (TIF) [file pone.0125881.s001.tif]

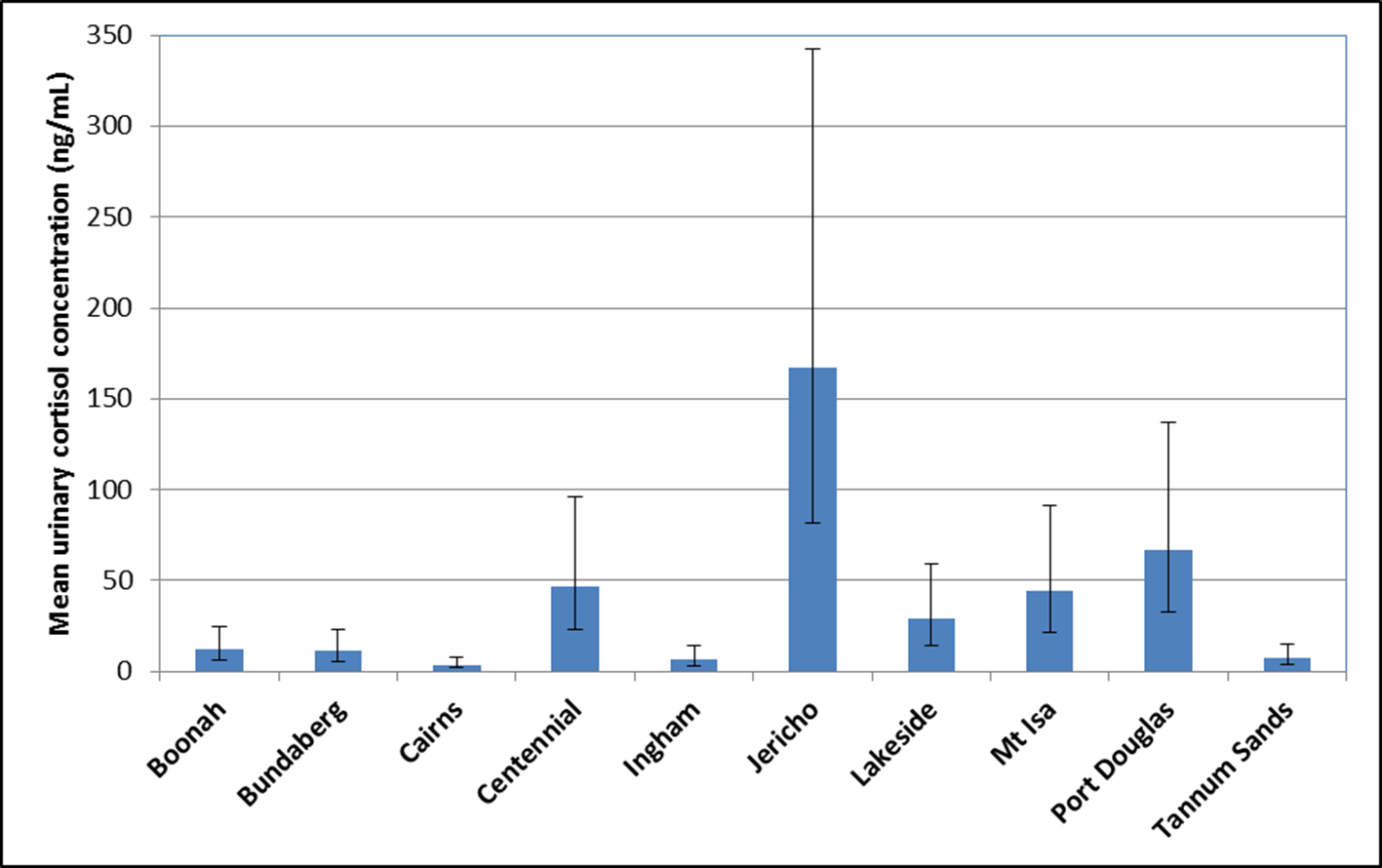

Supplement: S2 Fig — Error bars represent the mean ± one standard error, obtained by back-transforming variance from the logistic scale. Y axis scales are the same as Fig 2B to facilitate direct comparison with roosts subject to permitted disturbance. [Note ‘Lakeside’ = ‘Yungaburra’]. (TIF) [file pone.0125881.s002.tif]
