# Supplementary material for: Flying-Fox Roost Disturbance and Hendra Virus Spillover Risk
Source: PLoS One. 2015 May 27;10(5):e0125881. doi: 10.1371/journal.pone.0125881 (PMC4446312; doi:10.1371/journal.pone.0125881)
Supplement: S1 Table — (DOCX) [file pone.0125881.s003.docx]

| S1 Table: Geographic and DMP detail of 21 flying-fox roosts monitored in the eastern Australian states of Queensland and New South Wales between September 2011 and November 2012. | | | | | | |
| --- | --- | --- | --- | --- | --- | --- |
| Location | State | Description | Species present^1^ | DMP Application status | Disturbance commenced | Disturbance  ceased |
| Primary roosts |  |  |  |  |  |  |
| Barcaldine | QLD | Rural town | r | Approved 25/08/11 | 05/09/11 | 10/09/11 |
| Gayndah (2011) | QLD | Rural town | br | Approved 12/08/11 | 25/09/11 | 06/10/12 |
| Gayndah (2012) | QLD | Rural town | br | Approved 12/08/11^2^ | 03/02/12 | 19/03/12 |
| Sydney (RBG) | NSW | Capital city | bg | Approved 05/08/09 | 04/06/12 | 31/12/12 |
| Charters Towers | QLD | Rural city | br | Approved 21/02/12 | 04/06/12 | 31/08/12 |
| Duaringa | QLD | Rural town | r | Approved 17/05/12 | 07/10/12 | 15/10/12 |
| Jericho | QLD | Rural town | r | Approved 10/01/12^3^ | NA | NA |
| Yungaburra | QLD | Rural town | s | Approved 06/02/12^4^ | NA | NA |
| Cairns | QLD | Coastal city | rs | Refused 12/06/12 | NA | NA |
| Collinsville | QLD | Rural town | br | Withdrawn 09/01/12 | NA | NA |
| Boonah | QLD | Rural town | bg | Pending | NA | NA |
| Mt Isa | QLD | Rural city | r | Pending | NA | NA |
| Secondary roosts |  |  |  |  |  |  |
| Batemans Bay | NSW | Coastal town | g | NA | NA | NA |
| Blackbutt | NSW | Coastal city | g | NA | NA | NA |
| Bundaberg | QLD | Coastal city | bgr | NA | NA | NA |
| Sydney (CP) | NSW | Capital city | g | NA | NA | NA |
| Coulston Lakes | QLD | Rural village | b | NA | NA | NA |
| Great Keppel Is | QLD | Island | b | NA | NA | NA |
| Ingham | QLD | Rural town | bgrs | NA | NA | NA |
| Port Douglas | QLD | Coastal town | rs | NA | NA | NA |
| Tannum Sands | QLD | Coastal town | bgr | NA | NA | NA |
| Yeppoon | QLD | Coastal town | bgr | NA | NA | NA |
| ^1^ b = black flying-fox (*P. alecto*), g = grey-headed flying-fox (*P. poliocephalus*), r = little red flying-fox (*P. scapulatus*), s = spectacled flying-fox (*P. conspicillatus*). Species presence was constant over time at most roosts sampled on multiple occasions, though the relative proportion of each species sometimes varied. For Gayndah 2012, Charters Towers, Bundaberg, Ingham and Tannum Sands, species presence sometimes varied between sampling events, and for these roosts, the data is cumulative.’  ^2^ A second permit was issued on 10/02/2012 concurrent with the expiration of the original permit, hence the disturbance at Gayndah in 2012 was technically carried out under a separate permit.  ^3^ Permit granted for roost modification after flying-foxes moved of their own volition.  ^4^ Permit granted for dispersal or modification, but no such activity occurred within the study period as the flying-foxes moved of their own volition.  [Sydney (RBG) = Sydney Royal Botanic Gardens; Sydney (CP) = Sydney Centennial Park.] | | | | | | |
