# Supplementary material for: Flying-Fox Roost Disturbance and Hendra Virus Spillover Risk
Source: PLoS One. 2015 May 27;10(5):e0125881. doi: 10.1371/journal.pone.0125881 (PMC4446312; doi:10.1371/journal.pone.0125881)
Supplement: S2 Table — [The Gayndah roost had permitted disturbance events in 2011 and 2012. FF = flying-foxes, P = permitted disturbance activities, NP = non-permitted disturbance activities.] (DOCX) [file pone.0125881.s004.docx]

| S2 Table: Qualitative assessment of flying-fox distress associated with six permitted roost disturbance events in the eastern Australian states of Queensland and New South Wales between September 2011 and November 2012. [The Gayndah roost had permitted disturbance events in 2011 and 2012. FF = flying-foxes, P = permitted disturbance activities, NP = non-permitted disturbance activities.] | | | | | | | | |
| --- | --- | --- | --- | --- | --- | --- | --- | --- |
| **Roost** | **Date** | **Nature of activity** | **Timing** | **Flying-fox behaviour** | **Magnitude** | **Frequency** | **Assessed Impact** | **Overall Impact** |
| **Barcaldine** | Sep 2011 | Day-time disturbance of roosting FFs; noise, entering roost, vehicle horns (NP) | Pre-dispersal | Flight, confusion and eventual resettling of FFs within the roost | Moderate | Moderate | Moderate |  |
|  | Sep 2011 | Night-time removal of roost vegetation (P) | During dispersal | Confusion, circling, vocalisation of FF returning to roost | High | Low | High |  |
|  | Sep 2011 | Noise, smoke and lights directed at FFs returning to roost at dawn (P) | During dispersal | Confusion, circling, female FFs attempting to collect dependent young, roosting at alternative site | High | Low | High | Extreme |
|  | Sep 2011 | Daytime shooting at FFs attempting to roost at alternative site (NP) | During dispersal | Flight, confusion, circling, preventing FFs from taking up alternative roost sites at completion of permitted disturbance activities | Extreme | Low | Extreme |  |
|  | Sep 2011 | Daytime disturbance of roosting FFs; noise, entering roost, vehicle horns (NP) | During dispersal | Flight within the roost, preventing FFs from taking up alternative roost sites at completion of permitted disturbance activities | High | Low | High |  |
| **Gayndah** | Sep - Nov 2011 | Dusk gas-gun discharge (NP) | Pre-dispersal, during dispersal | Minimal effect as directed at FFs at fly-out and activity across the river from roost | Low | High | Low |  |
|  | Sep 2011 - Apr 2012 | Daytime disturbance of roosting FFs; handclapping, rock throwing, vehicle horns, yelling, mowers, brush-cutters, chain saws (NP) | Pre-dispersal, during dispersal | Flight, confusion and eventual resettling of FFs within the roost | Moderate | High | Moderate | High |
|  | Sep - Oct 2011 | Night-time removal of roost vegetation (P) | During dispersal | Confusion, circling, vocalisation of FF on return to roost; roost in alternate roost trees over time, moving west along river to school and caravan park | High | Low | High |  |
|  | Feb - Mar 2012 | Noise, smoke and lights directed at FFs returning to roost at dawn (P) | During dispersal | Confusion, circling, vocalisation, reluctance to roost in alternate roost trees or return to favoured roost trees. | High | Moderate | High |  |
| **Sydney Royal Botanical Gardens** | Jun - Dec 2012 | Intermittent noise and lights directed at FFs returning to roost at dawn (P) | During dispersal | Some confusion, circling, vocalisation of FF on return to roost, interspersed with normal roosting behaviour during disturbance intermissions | Moderate | High | Low | Low |
| **Charters Towers** | Jul - Aug 2012 | Daytime removal and trimming of unoccupied trees previously damaged by roosting FFs, with 60m buffer zone delimiting work from current roost (P) | During dispersal | Minimal disturbance; transient lifting of small numbers of animals | Low | High | Low | Low |
| **Duaringa** | Mar 2012 | Daytime disturbance of roosting FFs soon after the arrival of FFs in township (NP) | Pre-dispersal | Fragmentation of roost structure; 2/3 roost remained in public park, 1/3 relocated to the yards of a small number of adjacent residential homes | High | Low | High |  |
|  | May 2012 | Daytime disturbance of FFs roosting in public park (NP) | Pre-dispersal | Departure of all remaining FFs from public park | High | Low | High | High |
|  | Oct 2012 | Night-time removal of roost vegetation (P) | During dispersal | Returning FFs relocated to alternate roost trees in public park | High | Low | High |  |
|  | Oct-12 | Noise, smoke and lights directed at FFs returning to roost at dawn (P) | During dispersal | Over period of a week returning FFs relocated to alternate roost trees in quarry on the outskirts of township | High | Low | High |  |
